# Effectiveness of a Nutritional Intervention in Patients with Chronic Heart Failure at Risk of Malnutrition: A Prespecified Subanalysis of the PACMAN-HF Trial

**DOI:** 10.3390/nu17172899

**Published:** 2025-09-08

**Authors:** Carolina Ortiz-Cortés, Purificación Rey-Sánchez, Paula Gómez-Turégano, Ramón Bover-Freire, Julián F. Calderón-García, Jose Javier Gómez-Barrado, Sergio Rico-Martín

**Affiliations:** 1 Cardiology Department, Hospital Universitario Fundación de Alcorcón, 28922 Madrid, Spain; 2 Department of Nursing, Colegio de Enfermería y Terapia Ocupacional, Universidad de Extremadura, 10003 Cáceres, Spain; prey@unex.es (P.R.-S.);; 3 Cardiology Department, Hospital Universitario San Pedro de Alcántara, 10001 Cáceres, Spain; paula.gt@hotmail.es (P.G.-T.); jjgomezbarrado@gmail.com (J.J.G.-B.); 4 Cardiology Department, Hospital Universitario Clínico San Carlos, 28040 Madrid, Spain; ramonboverfreire@gmail.com

**Keywords:** heart failure, risk of malnutrition, prognosis, nutritional intervention, functional capacity

## Abstract

**Background and objectives**: Nutritional disorders are common in patients with heart failure (HF) and are associated with reduced functional capacity and poor prognosis. In this study, we evaluated the prognostic, nutritional and functional impact of a structured nutritional intervention in patients with chronic HF at risk of malnutrition. **Methods**: This is a prespecified subanalysis of the randomized controlled trial *Prognostic And Clinical iMpAct of a Nutritional intervention in patients with chronic HF (PACMAN-HF)*. Ambulatory patients with chronic HF at risk of malnutrition were identified using the Mini Nutritional Assessment (MNA) questionnaire and randomized to receive either an individualised nutritional intervention (intervention group) or standard care (control group). We evaluated the frequency of malnutrition risk and the impact of the intervention on clinical outcomes, defined as a composite of all-cause mortality or time to first HF hospitalisation, as well as nutritional status and functional capacity at 3- and 12-month follow-ups. **Results**: A total of 225 patients were screened. Of these, 72 (32%) were identified as being at risk of malnutrition and 64 (28.4%) met the inclusion criteria and were randomized (31 in the intervention group and 33 in the control group). There were no significant differences between the groups in terms of all-cause mortality or time to first HF hospitalisation (HR = 0.34 [0.11–1.09]; *p* = 0.072). At 12 months, the intervention group demonstrated a significant improvement in functional capacity, with an increase of 31.3 metres in the 6-minute walk test (6MWT) (*p* = 0.002), whereas no significant change was observed in the control group. Nutritional status improved significantly in the intervention group (MNA score +4.12, *p* < 0.001) and declined in the control group (−1.15, *p* = 0.029). At 12 months, body mass index, tricipital skinfold thickness, arm circumference, and serum albumin levels increased in the intervention group. **Conclusions**: A structured and individualised nutritional intervention significantly improved nutritional status and functional capacity over 12 months, although it did not impact major clinical outcomes.

## 1. Introduction

Heart failure (HF) is a multifaceted and progressive clinical syndrome that persists as a major global health challenge [1,2,3]. Undernutrition in HF is highly prevalent and increases with age, with reported rates varying across series from 15% to 90%, depending on disease severity and the assessment method used [4,5,6]. It is associated with worse disease progression, greater impairment in functional capacity and quality of life, increased risk of rehospitalisation, longer hospital stays and increased mortality [7,8,9]. Previous research has revealed an inverse relationship between the degree of malnutrition and prognosis in HF patients, indicating that even in less advanced stages of malnutrition, there is increased mortality [9,10,11,12,13,14]. Specifically, compared with patients with normal nutritional status, the risk of malnutrition doubles the risk of mortality, as assessed by the Mini Nutritional Assessment (MNA) score [9]. Furthermore, mortality is again doubled in patients with more severe malnutrition compared to those at risk of malnutrition.

Similar results have been reported regarding the impact of nutritional alterations on functional capacity, with worse results in functional tests both in malnourished patients and in patients at risk of malnutrition than in a population with normal nutritional status [15,16]. These findings underscore the importance of early detection and management of nutritional deficits, even at the earliest stages.

Despite the relevance of these data, most existing studies have focused on patients with acute HF and established malnutrition. Therefore, the objective of this study was to assess the prognosis and nutritional and functional impact of a structured nutritional intervention in patients with chronic HF at risk of malnutrition. 

## 2. Materials and Methods

### 2.1. Study Design and Population

The study “*Prognostic And Clinical iMpAct of a Nutritional intervention in patients with chronic HF (PACMAN-HF)*” is a single-centre, randomized, controlled clinical trial. The study design, randomization and blinding process, baseline characteristics, and primary results have been previously published [17]. Briefly, PACMAN-HF was conducted between January 2018 and January 2021. A total of 225 patients with chronic HF were screened, of whom 86 with malnutrition or at risk of malnutrition were randomized in a 1:1 ratio to an individualized nutritional intervention (*n* = 42) or conventional management (*n* = 44). Patients were followed for 12 months. The primary endpoint was a composite of all-cause mortality or time to first HF hospitalization, and the secondary endpoints included changes in nutritional status and functional capacity.

**Figure 1:**
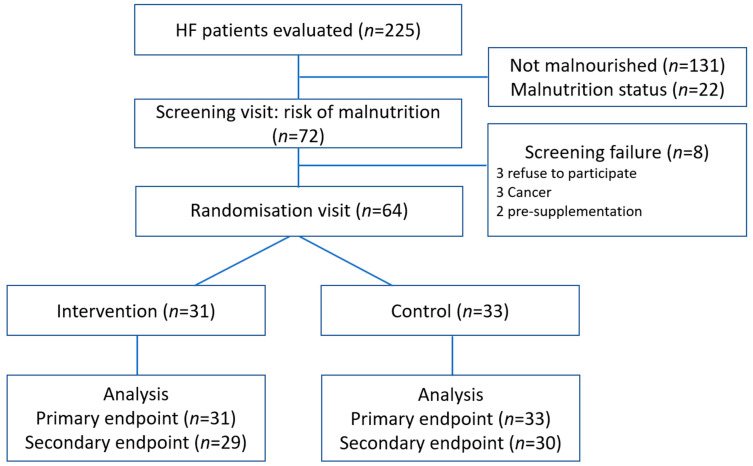
Flowchart of study patients. HF, Heart failure.

The current work represents a prespecified sub-study including patients aged 18 years and older with a confirmed diagnosis of chronic HF according to the clinical practice guidelines in force at the start of the study [18] (defined by the presence of signs and/or symptoms of HF, together with objective evidence of structural abnormality [imaging and/or cardiac biomarkers], irrespective of left ventricular ejection fraction). The analysis focuses on patients who were at risk of malnutrition, as identified by the MNA questionnaire score between 17 and 23.5. In this population, we specifically assessed the effect of the nutritional intervention on nutritional status (MNA score and anthropometric/biochemical parameters) and functional capacity (6 min walk test [6MWT] and NYHA functional class) at 3 and 12 months, and on clinical outcomes (all-cause mortality and HF hospitalizations) at 12 months of follow-up. The study flow chart is summarized in Figure 1, whereas the detailed timeline of visits and assessments is provided in Table 1.

Patients with established malnutrition (MNA < 17), as well as those who were unable to complete the 6MWT, with advanced cognitive impairment, active cancer, end-stage comorbidities, pregnancy, or prior nutritional supplementation, were excluded.

All patients agreed to participate and signed the informed consent form. This study was approved by the research committee of the Hospital Universitario San Pedro de Alcántara from Cáceres and registered in ClinicalTrials.gov (NCT05527522).

The included patients were randomized to receive an individualised nutritional intervention associated with conventional management (intervention group) versus conventional management (control group).

### 2.2. Intervention, Study Visits and Procedures

Participants in the control and intervention groups received standard HF therapy in accordance with the most up-to-date clinical practice guidelines available at the initiation of the study [18]. The personalised dietary plan was designed in accordance with energy intake guidelines and nutritional objectives while also being tailored to the patient’s comorbid conditions. The development of the individualised diet followed these steps:

1. During the clinical interview, an estimation of the patient’s energy intake and dietary composition was obtained through a 24 h dietary recall, which was conducted by trained research nurses using a structured interview format based on a validated questionnaire. Dietary intake was recorded on three nonconsecutive days (two weekdays and one weekend day) to better reflect the patients’ usual dietary habits.
2. Nutritional requirements were individually assessed using the outpatient nutritional needs calculator provided by the Spanish Society of Endocrinology and Nutrition [19].
3. On the basis of the comparison between actual intake and estimated requirements, a personalised dietary regimen was formulated, considering relevant comorbidities—primarily diabetes mellitus and chronic kidney disease. The plan was generated using Dietopro^®^ software, version 1.0, a diet-therapy management software developed in Spain that facilitates the creation of individualized weekly meal plans aligned with nutritional requirements [20]. The proposed plans were subsequently reviewed and adjusted by the clinical research team according to patient comorbidities and guideline-based recommendations. The diet was adjusted according to the following criteria: an energy intake of 27 kcal/kg/day for non-obese participants, with a 30% reduction in those with BMI > 30 kg/m^2^, and a protein intake of 1.5 g/kg/day for patients with normal renal function. In patients with renal impairment (defined as eGFR < 60 mL/min/1.73 m^2^), protein intake was reduced to approximately 0.8–1.0 g/kg/day [21].

Nutritional status was assessed using the MNA questionnaire, which comprises 18 items grouped into four domains: anthropometric measurements, general health status, dietary information, and subjective self-assessment [22]. Patients were classified into three categories according to their total score: well nourished (score ≥ 24), at risk of malnutrition (score 17–23.5), and malnourished (score < 17). The MNA was administered at baseline and again at the end of the follow-up period. Only patients classified as being at risk of malnutrition were eligible for inclusion in the study.

To complement the nutritional assessment, both biochemical and anthropometric parameters were collected. Anthropometric evaluations included body mass index (BMI), waist circumference (WC), hip circumference (HC), arm circumference (AC), calf circumference (CC), and skinfold thickness (total fold, TF). The analytical parameters included complete blood count, coagulation panel, renal and hepatic function tests, lipid profile, and glucose metabolism markers.

Functional capacity was evaluated using the 6MWT, which was performed at baseline and subsequently at 3 and 12 months [23]. The total distance walked was recorded at each point.

### 2.3. Statistical Analysis

The data were analysed on an intention-to-treat basis. All the statistical analyses were performed with SPSS v.27. Continuous variables are presented as the means ± standard deviations, and categorical variables are presented as frequencies (%). A normal distribution was considered when *p* > 0.05 according to the Kolmogorov–Smirnov test. Participants in the control group and intervention group were compared for continuous variables by Student’s *t* test (if normally distributed) or the Mann–Whitney U test (if not normally distributed). Qualitative variables were compared by *X*^2^ tests or Fisher’s exact tests if necessary. Student’s test for paired samples (if normally distributed) or the Wilcoxon test (if nonnormally distributed) were used to detect changes in both the control and intervention groups between baseline and 3 months or 12 months. Kaplan–Meier curves of the time to manifestation of the primary composite outcome were plotted for each group and compared using the log-rank test. Multivariate adjustment was performed using Cox regression and included the age (years) and functional capacity (6MWT) and the following categorical covariates: sex (male or female), diabetes mellitus status (yes or no), renal function status (yes or no) and NYHA functional class (I, II, III or IV).

## 3. Results

A total of 225 patients were initially screened, of whom 72 (32%) were identified as being at risk of malnutrition according to the MNA (score 17–23.5). Eight did not meet the inclusion criteria and were therefore excluded. The remaining 64 patients provided informed consent and were randomized: 31 to the intervention group and 33 to the control group. A comprehensive flowchart illustrating patient selection and follow-up is shown in Figure 1. During the study period, 5 patients died (2 in the intervention group and 3 in the control group) resulting in 59 patients completing the study (29 in the intervention group and 30 in the control group).

### 3.1. Baseline Characteristics

The baseline characteristics are summarised in Table 2. The mean age was 72.07 ± 11.16 years, and 43 participants (67.18%) were male. The comorbidities included hypertension in 39 patients (60.93%), dyslipidaemia in 35 (54.68%), type 2 diabetes in 21 (32.81%), and a history of smoking in 31 (48.43%). HF was of ischaemic origin in 30 patients (46.87%). The mean left ventricular ejection fraction (LVEF) was 31.90 ± 7.69%. According to the New York Heart Association (NYHA) classification, 54.68% of the patients were in functional class II, and 26.56% were in class III. The mean baseline distance on the 6MWT was 338.78 ± 91.50 m. The baseline characteristics and medical treatments were well balanced between the two groups, except to hypertension where control group vs. intervention group had more hypertensive patients (79.3% vs. 51.61%; *p* = 0.027).

### 3.2. Primary Endpoint: Effects of Nutritional Intervention on Prognosis

**Figure 2:**
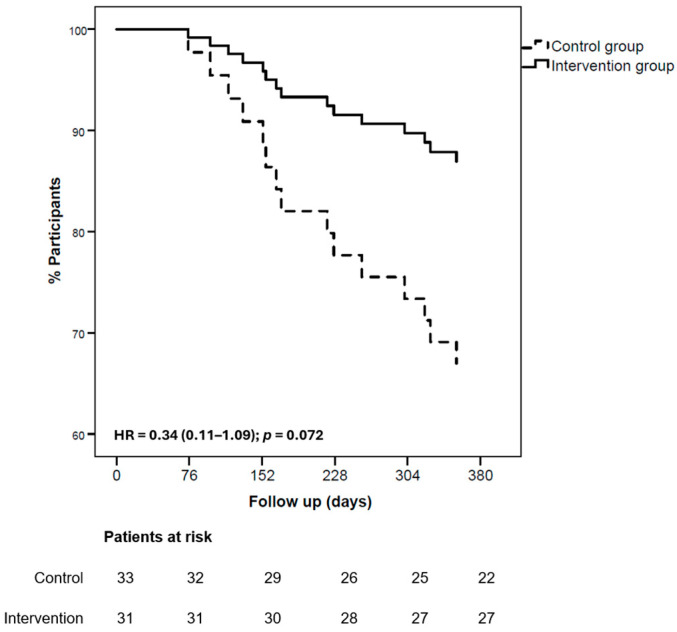
Kaplan–Meier curves for the primary outcome (composite of all-cause mortality or first HF hospitalization). Hazard ratios are from Cox models adjusted for age, sex, diabetes mellitus, renal dysfunction, baseline 6MWT distance, and NYHA class.

**Figure 3:**
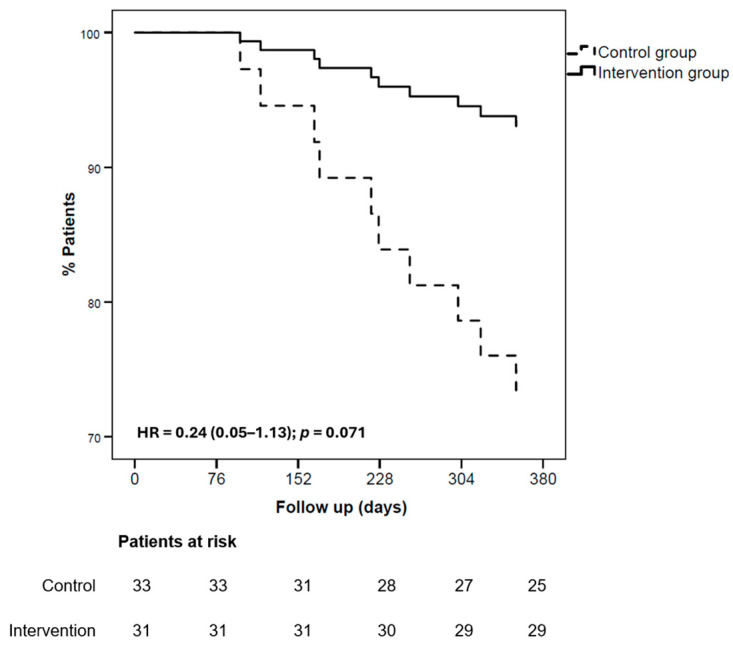
Kaplan–Meier curves for time to first HF hospitalization. Hazard ratios are from Cox models adjusted for age, sex, diabetes mellitus, renal dysfunction, baseline 6MWT distance, and NYHA class.

At the 365-day follow-up (Figure 2), the intervention had no statistically significant effect (HR = 0.34 (0.11–1.09); *p* = 0.072). The primary endpoint (all-cause mortality or first HF hospitalisations) occurred in 4 patients in the intervention group (12.9%) and 11 in the control group (33.3%). This non-significant benefit was related mainly to the lower number of HF hospitalisations in the intervention group (Figure 3): 2 patients (6.4%) versus 8 patients (24.4%) in the control group (HR = 0.24; 95% CI = 0.05–1.13; *p* = 0.071). All-cause mortality occurred in 2 patients (6.4%) in the intervention group and in 3 patients (8.9%) in the control group.

### 3.3. Secondary Endpoints: Effects of Nutritional Intervention on Nutritional Status and Functional Capacity

The impact of the intervention on nutritional status and functional capacity was evaluated at the 3- and 12-month follow-ups among the 59 surviving patients (control group: *n* = 30; intervention group: *n* = 29). In terms of nutritional status, the intervention group experienced a significant improvement in the MNA score, which increased from 20.1 to 24.22 points at 12 months (Δ 4.12 points; 95% CI 3.1–5.2; *p* < 0.001). Conversely, the control group showed a significant decrease in the MNA score from 20.31 to 19.16 (Δ –1.15 points; 95% CI 0.12–2.18; *p* = 0.029). In addition, statistically significant differences were observed when comparing the effects of the intervention between the two groups (*p* < 0.001). At the end of follow-up, 9 patients (31.03%) in the intervention group achieved normalisation of their nutritional status, whereas only 1 patient (3.3%) in the control group achieved normalisation. (*p* < 0.01).

In terms of analytical and anthropometric parameters, the intervention group presented significant increases in BMI and serum albumin levels, as well as improvements in tricipital skinfold thickness and arm circumference at 12 months. Except for the increase in albumin—which reached statistical significance at 3 months—all the other improvements were observed only at 12 months. In contrast, no significant changes in these parameters were observed in the control group throughout the study period.

The effects of nutritional intervention on nutritional status and function are detailed in Table 3. At 12 months, the intervention group presented a significant increase in the 6MWT distance from 354 metres at baseline to 385 metres (Δ 31.3 m; 95% CI 10.3–52.3; *p* = 0.002), whereas no significant change was observed in the control group. At 3 months, no significant differences in the 6MWT distance were observed between the two groups.

On the other hand, when we compared the effects obtained between both groups we observed statistically significant differences in serum album levels, total cholesterol, HDL and serum transferrin levels at 3 months follow-up. After 12 months of follow-up, statistically significant differences were observed between the effects of both groups in the MNA, BMI, triceps skinfold and 6MWT values.

## 4. Discussion

Unlike most published studies, which have focused on hospitalised patients with established malnutrition, our study addresses a population at the early stages of nutritional decline, representing a meaningful opportunity for preventive interventions. Notably, one of the key findings was the normalisation of nutritional status in a considerable proportion of patients in the intervention group (31%) compared with (3.3%) in the control group. This result suggests that a structured and individualised approach can reverse nutritional risk in an outpatient population before malnutrition becomes established. Future studies should explore whether this early recovery is associated with long-term clinical benefits.

In terms of clinical outcomes, no significant differences were observed between groups in the composite of all-cause mortality or time to first HF hospitalisation, in contrast to what was observed in the overall population of the PACMAN-HF study or in previous studies [9,24]. This lack of effect may be due to several factors, including the reduced sample size of this subgroup, the lower baseline clinical risk, and the limited duration of follow-up. Nonetheless, relevant benefits were observed in terms of nutritional and functional parameters. The intervention was associated with significant improvements in nutritional status, as evidenced by increases in the MNA score, body mass index and tricipital skinfold thickness. These improvements were significant at 12 months but not earlier, underscoring the need for sustained interventions to achieve clinically meaningful changes.

From a functional perspective, at 12 months the intervention group showed a significant improvement in exercise capacity, with an increase of 31.3 m in the distance covered during the 6MWT, compared with no change in the control group. This difference was both statistically significant and clinically relevant, as the 6MWT distance correlates with quality of life, functional status, and prognosis in HF patients.

Published results on the impact of nutritional interventions on functional capacity and nutritional status in patients with HF have been inconsistent, and studies specifically targeting populations at risk of malnutrition are scarce. A meta-analysis [25] reported a significant increase in body weight (+3.83 kg; 95% CI: 0.17–7.50; *p* = 0.04) following nutritional intervention in HF patients who were malnourished or at risk of malnutrition, although no significant changes were observed in triceps skinfold thickness.

Baseline characteristics were largely balanced; the only difference was a slightly higher prevalence of hypertension in the control arm. Because endpoints were analyzed as change from baseline, with comparable starting values and a consistent pattern of benefit in the intervention group, this minor imbalance is unlikely to explain the findings. This sub-study did not prespecify or evaluate traditional cardiovascular risk factors (e.g., blood pressure) and was not intended to assess risk factor modification; therefore, no conclusions on risk factor control can be drawn.

Another relevant contribution is the fact that such a high prevalence of nutritional disorders was detected in cardiology outpatient clinics—where it might be considered less likely due to the younger age and lower comorbidity burden of patients—and the demonstration of the feasibility and clinical utility of implementing personalized nutritional interventions in this setting. In this regard, telemedicine could be a particularly useful tool, as it facilitates periodic assessment and reinforcement of dietary habits and has been associated with reductions in HF hospitalizations and, in some programs, mortality [26,27].

Taken together, the findings of this subanalysis reinforce the need to incorporate nutritional assessment and management into the comprehensive care of patients with chronic HF, even in the early stages of deterioration. Although no differences were found in major clinical events in this study, the improvements observed in nutritional status and functional capacity justify the implementation of targeted nutritional strategies and pave the way for future studies with greater statistical power and longer follow-up periods.

This study has several limitations. First, the sample size of the subgroup analysed was relatively small, which may have limited the statistical power to detect differences in clinical events. Second, it was conducted at a single centre, which may reduce the generalisability of the findings. Third, although the follow-up duration was sufficient to observe changes in nutritional and functional parameters, it may have been too short to assess potential long-term effects on morbidity and mortality. Finally, relevant geriatric syndromes such as frailty and sarcopenia—which are closely related to nutritional status and prognosis in HF—were not assessed, potentially omitting important contributors to the observed outcomes.

## 5. Conclusions

In conclusion, a structured and individualised nutritional intervention significantly improved nutritional status and functional capacity in patients with HF and risk of malnutrition, although it did not impact major clinical outcomes. These findings highlight the importance of early diagnosis and management of nutritional disorders in patients with HF.

## Figures and Tables

**Table 1:** Timeline of study.

	Month 0	Month 1	Month 3	Month 6	Month 9	Month 12	Post-Study
MNA	X					X	
Inclusion	X						
Randomisation	X						
Clinical review	X	X	X		X	X	
Phone contact				X			
Nutritional intervention *	X	X	X	X	X	X	
Anthropometrics measurements	X		X			X	
Blood sample	X		X			X	
6MWT	X		X			X	
Statistical analysis							X

* The nutritional intervention was only carried out in the intervention arm. Abbreviations: 6MWT, 6 min walking test; MNA, mini nutritional assessment.

**Table 2:** Baseline characteristics of participants.

	All Patients (*n* = 64)	Control (*n* = 33)	Intervention (*n* = 31)	*p*-Value
Age (years)	72.07 ± 11.16	70.83 ± 11.80	73.24 ± 10.57	0.394
Men (%)	43 (67.18)	22 (66.66)	21 (67.64)	0.927
Hypertension (%)	39 (60.93)	23 (79.30)	16 (51.61)	0.027
Dyslipidaemia (%)	35 (54.68)	19 (57.57)	16 (51.86)	0.410
Diabetes Mellitus (%)	21 (32.81)	13 (39.39)	8 (25.8)	0.187
History of smoking (%)	31 (48.43)	15 (45.45)	16 (51.61)	0.404
Ischaemic heart disease (%)	30 (46.87)	15 (45.45)	15 (48.38)	0.506
Atrial fibrillation (%)	32 (50.0)	17 (51.51)	15 (48.38)	0.451
eGFR < 60 mL/min/1.73 m^2^ (%)	39 (60.93)	19 (57.57)	20 (64.51)	0.378
EF (%)	31.90 ± 7.69	32.28 ± 8.75	31.51 ± 6.53	0.696
Functional class				
NYHA I (%)	8 (12.5)	4(12.12)	4(12.9)	0.761
NYHA II (%)	35 (54.68)	21 (63.63)	14 (45.16)	0.067
NYHA III (%)	17 (26.56)	7 (21.21)	10 (32.25)	0.553
NYHA IV(%)	4 (6.25)	1 (3.03)	3(9.6)	0.124
MNA score	20.26 ± 1.84	20.28 ± 1.77	20.24 ± 1.95	0.925
NTproBNP, pg/mL	2401.28 ± 2042.69	2306.28 ± 1879.73	2506.46 ± 2239.74	0.711
Serum sodium, mmol/L	140.06 ± 3.27	140.50 ± 3.61	139.60 ± 2.85	0.283
Serum potassium mmol/L	4.78 ± 0.79	4.73 ± 0.39	4.84 ± 1.07	0.567
Albumin, g/dL	3.93 ± 0.50	4.00 ± 0.46	3.86 ± 0.53	0.262
Total cholesterol, mg/dL	138.35 ± 28.61	141.06 ± 25.54	135.48 ± 31.73	0.440
HDL, mg/dL	46.82 ± 14.24	47.24 ± 15.44	46.38 ± 13.08	0.812
LDL, mg/dL	69.04 ± 27.32	68.42 ± 21.08	69.79 ± 33.06	0.853
Haemoglobin, g/dL	13.45 ± 1.80	13.31 ± 1.52	13.62 ± 2.07	0.509
Lymphocytes, ×10^9^/L	1.79 ± 0.76	1.77 ± 0.61	1.80 ± 0.88	0.844
Transferrin, mg/dL	233.23 ± 43.99	226.04 ± 50.95	239.97 ± 35.78	0.208
BMI kg/m^2^	26.56 ± 4.11	27.32 ± 4.01	25.75 ±4.13	0.127
Waist circumference, cm	102.31 ± 12.36	104.27 ± 12.05	100.22 ± 12.55	0.193
Arm circumference, cm	27.53 ± 3.91	27.60 ± 4.53	27.45 ± 3.20	0.876
Hip circumference, cm	101.62 ± 8.98	102.18 ± 10.15	101.03 ± 7.67	0.613
Calf circumference, cm	33.90 ± 3.21	33.70 ± 3.68	34.01 ± 3.66	0.213
Tricipital fold, mm	13.21 ± 2.27	13.39 ± 2.13	13.03 ± 2.44	0.503
6MWT, m	338.78 ± 91.50	330.78 ± 94.35	347.29 ± 89.11	0.475

Abbreviations: eGFR, estimated glomerular filtration Rate; EF, ejection fraction; NYHA, New York heart association; MNA, mini nutritional assessment; NTproBNP, *N*-terminal pro-brain natriuretic peptide; HDL, high-density lipoprotein; LDL, low-density lipoprotein; BMI, body mass index; 6MWT, 6 min walking test.

**Table 3:** Effect of the intervention on nutritional status and functional capacity at 3- and 12-month follow-up.

		Baseline	At 3 Months			At 12 Months		
	Group ^†^	Mean ± SD	Mean ± SD	∆ *p*-Value *	*p*-Value ^‡^	Mean ± SD	∆ *p*-Value *	*p*-Value ^‡^
MNA	Control	20.31 ± 1.84	-	-	-	19.16 ± 2.84	−1.15 ± 3.18 0.029	<0.001
	Intervention	20.10 ± 1.79	-	-		24.22 ± 2.78	4.12 ± 2.36 <0.001	
Albumin, g/dL	Control	4.03 ± 0.47	4.03 ± 0.42	0.00 ± 0.49 0.957	0.038	3.95 ± 0.44	0.08 ± 0.61 0.498	0.277
	Intervention	3.85 ± 0.52	4.11 ± 0.40	0.25 ± 0.41 0.002		4.08 ± 0.39	0.23 ± 0.42 0.008	
Total cholesterol, mg/dL	Control	138.56 ± 23.09	141.56 ± 23.06	3.00 ± 16.60 0.331	0.037	138.88 ± 24.49	0.32 ± 6.14 0.958	0.056
	Intervention	132.60 ± 30.91	127.17 ± 28.42	−5.42 ± 13.96 0.049		127.00 ± 29.06	−5.60 ± 16.05 0.076	
HDL mg/dL	Control	45.06 ± 13.20	48.53 ± 12.08	3.46 ± 4.65 <0.001	0.011	47.13 ± 12.16	2.06 ± 4.46 0.017	0.420
	Intervention	46.68 ± 13.49	46.41 ± 10.67	−0.27 ± 6.23 0.813		47.55 ± 12.23	0.86 ± 6.64 0.490	
LDL, mg/dL	Control	67.43 ± 20.39	69.06 ± 26.13	1.63 ± 12.24 0.471	0.063	66.60 ± 18.82	−0.83 ± 11.13 0.685	0.509
	Intervention	68.79 ± 33.34	63.00 ± 24.31	−5.79 ± 16.77 0.073		64.41 ± 25.47	−4.37 ± 26.43 0.380	
Hb g/dL	Control	13.31 ± 1.54	13.36 ± 1.55	0.05 ± 0.93 0.772	0.913	13.23 ± 1.73	−0.08 ± 1.04 0.666	0.758
	Intervention	13.67 ± 2.09	13.58 ± 1.82	0.08 ± 1.26 0.715		13.73 ± 1.81	0.06 ± 2.18 0.886	
Lymphocytes ×10^9^/L	Control	1.80 ± 0.90	2.00 ± 0.93	0.20 ± 0.35 0.004	0.542	1.84 ± 0.90	0.16 ± 0.40 0.031	0.308
	Intervention	1.82 ± 0.59	1.96 ± 0.66	0.13 ± 0.51 0.158		1.97 ± 0.67	0.15 ± 0.51 0.124	
Transferrin, mg/dL	Control	239.24 ± 36.52	233.68 ± 34.65	−5.56 ± 27.80 0.282	0.038	218.46 ± 62.72	−20.77 ± 60.37 0.070	0.975
	Intervention	225.43 ± 52.65	247.29 ± 37.29	21.85 ± 63.32 0.074		246.62 ± 41.49	−21.19 ± 64.62 0.127	
BMI, kg/m^2^	Control	27.48 ± 3.80	27.45 ± 3.89	−0.03 ± 1.29 0.884	0.297	27.73 ± 4.06	0.24 ± 1.63 0.417	0.044
	Intervention	25.69 ± 4.23	25.99 ± 3.82	0.30 ± 1.11 0.157		26.79 ± 4.06	1.10 ± 1.87 0.004	
Waist circumference, cm	Control	105.10 ± 10.84	105.70 ± 10.51	0.60 ± 3.16 0.308	0.462	105.40 ± 10.43	0.30 ± 4.61 0.725	0.907
	Intervention	100.03 ± 12.96	100.03 ± 12.83	0.00 ± 3.06 0.999		100.17 ± 13.15	0.13 ± 6.43 0.909	
Arm circumference, cm	Control	28.33 ± 4.04	28.73 ± 4.26	0.40 ± 1.13 0.063	0.386	28.63 ± 4.18	0.30 ± 1.66 0.332	0.095
	Intervention	27.62 ± 3.24	28.55 ± 3.31	0.93 ± 3.06 0.114		29.28 ± 4.63	1.66 ± 3.96 0.032	
Hip circumference, cm	Control	102.80 ± 10.21	103.26 ± 10.71	0.46 ± 4.36 0.563	0.662	102.86 ± 10.89	0.06 ± 3.32 0.913	0.189
	Intervention	101.24 ± 7.87	101.31 ± 7.97	0.06 ± 2.37 0.877		102.58 ± 8.00	1.34 ± 4.06 0.086	
Calf circumference, cm	Control	33.61 ± 2.96	34.06 ± 3.12	0.45 ± 1.51 0.114	0.980	33.93 ± 3.32	0.31 ± 1.24 0.177	0.810
	Intervention	34.51 ± 2.93	34.98 ± 2.53	0.46 ± 1.66 0.144		34.92 ± 3.10	0.41 ± 1.87 0.248	
Tricipital fold, mm	Control	13.53 ± 2.14	13.66 ± 2.05	0.13 ± 0.62 0.255	0.740	13.68 ± 2.07	0.15 ± 0.63 0.196	0.043
	Intervention	13.20 ± 2.42	13.28 ± 2.39	0.07 ± 0.76 0.582		14.01 ± 2.29	0.80 ± 0.99 <0.001	
6MWT	Control	334.33 ± 94.61	337.70 ± 105.36	3.33 ± 45.70 0.690	0.682	327.40 ± 110.44	−6.93 ± 42.91 0.383	0.002
	Intervention	354.03 ± 87.62	362.93 ± 91.30	8.89 ± 57.54 0.412		385.31 ± 72.62	31.30 ± 48.62 0.002	

^†^ 59 surviving patients (control group: *n* = 30; intervention group: *n* = 29); * Differences between baseline and at 3 or 12 months within the group; ^‡^ Differences in the effect of the intervention group vs. the control group. ∆ Changes between baseline and 3 or 12 months. Abbreviations: 6MWT, 6 min walking test; BMI, body mass index; Hb, haemoglobin; HDL, High-density lipoprotein; LDL, low-density lipoprotein; MNA, mini nutritional assessment.

## Data Availability

The data presented in this study are available on request from the corresponding author due to ethical restrictions.

## Abbreviations

The following abbreviations are used in this manuscript:

6MWT	6-Minute Walk Test
AC	Arm Circumference
BMI	Body Mass Index
CC	Calf Circumference
CI	Confidence Interval
HF	Heart Failure
HC	Hip Circumference
HR	Hazard Ratio
LVEF	Left Ventricular Ejection Fraction
MNA	Mini Nutritional Assessment
NYHA	New York Heart Association
PACMAN-HF	*Prognostic And Clinical iMpAct of a Nutritional intervention in patients with chronic HF*
TF	Total Skinfold Thickness
WC	Waist Circumference
